# Case Report: ECMO-Assisted Uniportal Thoracoscopic Tracheal Tumor Resection and Tracheoplasty: A New Breakthrough Method

**DOI:** 10.3389/fsurg.2022.859432

**Published:** 2022-04-04

**Authors:** Zi-Hao Li, Bo Dong, Chun-Li Wu, Shi-Hao Li, Bin Wu, Yin-Liang Sheng, Feng Li, Yu Qi

**Affiliations:** Department of Thoracic Surgery, The First Affiliated Hospital of Zhengzhou University, Zhengzhou, China

**Keywords:** thoracic surgery, tracheal stenosis, tracheal tumor resection, single-port thoracoscopic, ECMO-extracorporeal membrane oxygenation

## Abstract

Primary tracheal tumors are seldom seen, and most of them are malignant. At present, the main treatment is surgical resection. It is rare to accomplish tracheal tumor resection and tracheoplasty via uniportalal thoracoscopy. In order both to maintain the patient's oxygen supply during surgery and to reduce the size of the surgical incision, we have innovatively integrated the ECMO-assisted and uniportal thoracoscopic techniques for the first time, perfectly achieving tracheal tumor resection and tracheoplasty. The intraoperative manipulation was only 180 min in duration. The patient returned to the intensive care unit and recovered smoothly after the surgery. The patient was discharged from the hospital 17 days after the operation. ECMO-assisted uniportal thoracoscopic tracheal resection and tracheoplasty provides a new idea and method for colleagues.

## Introduction

Primary tracheal tumors are seldom seen, and most of them are malignant. The incidence of tracheal malignant tumors is ~0.1 for 100,000 persons per year, corresponding to 0.2% of all tumors of the respiratory tract (1). At present, the main treatment is surgical resection and tracheal reconstruction (2). Traditionally, right thoracotomy or median sternotomy has been used for tracheal resection and reconstruction. Another method developed in recent years is thoracoscopic surgery, which commonly requires 3 or more incisions (3–6).

The goal is to accomplish tracheal tumor resection and tracheoplasty with uniportal thoracoscopy. The primary technical difficulty is minimizing the influence of anesthetic endotracheal intubation and maintaining good ventilation function during surgery by establishing appropriate ventilation channels. In many cases, it is contradictory to guarantee adequate blood oxygenation and to gain excellent visualization of the surgical field. We thought extracorporeal membrane oxygenation (ECMO) might help us achieve the optimal surgical outcome (7). Finally, we innovatively integrated ECMO-assisted and uniportal thoracoscopic techniques for the first time, perfectly achieving simultaneous tracheal tumor resection and tracheoplasty.

## Methods

### Patient

The patient was a 50-year-old woman with initial symptoms of cough, expectoration and dyspnea. After anti-infective therapy in the local hospital, there was no obvious remission. Then, the patient was referred to our hospital for further treatment. She had no past history of smoking, cancer, cerebrovascular disease, hypertension and diabetes mellitus. Her pulmonary function test showed an FEV1/FVC ratio of 60%, an FEV1 of 68% predicted, and an FVC of 88% predicted.

**Figure 1:**
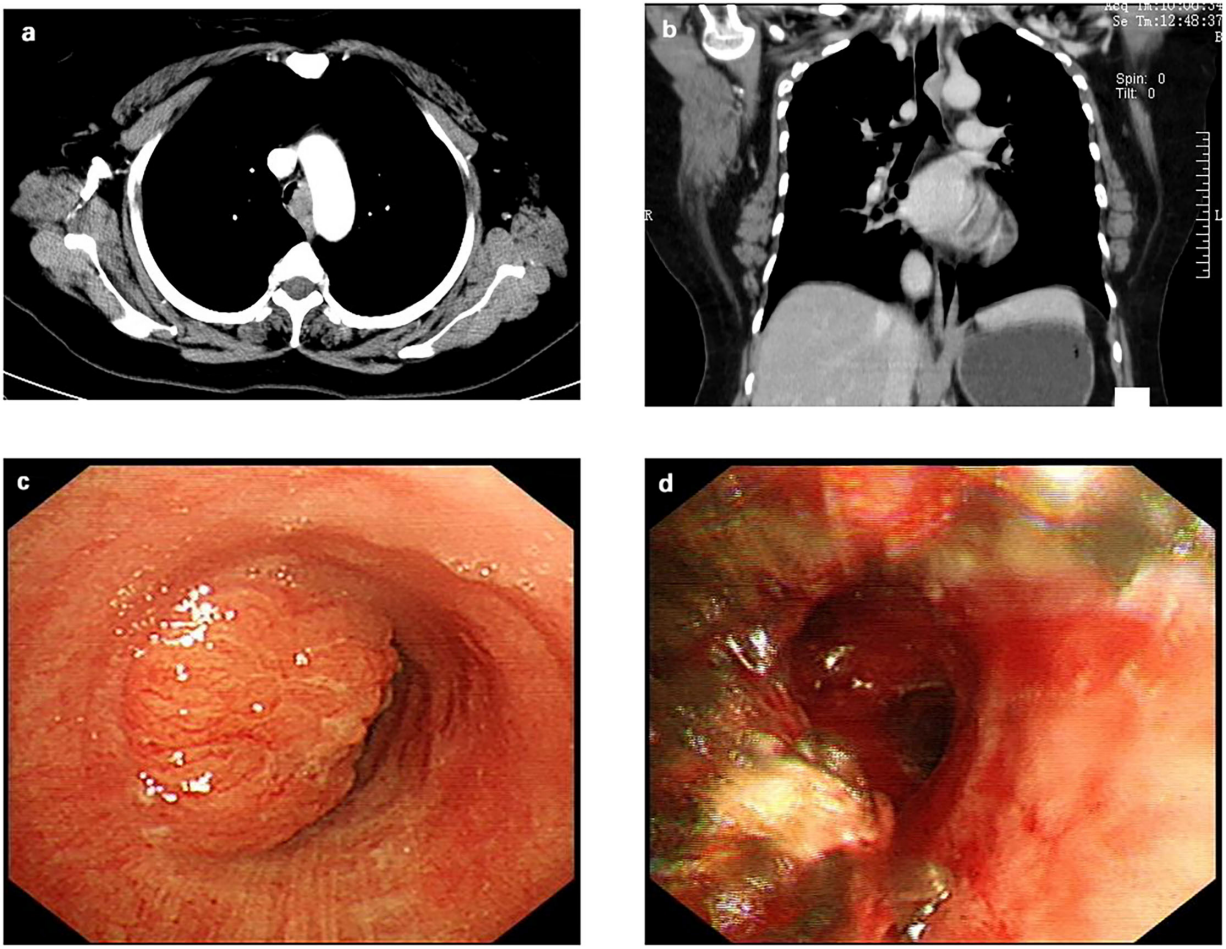
Preoperative examination of the patient. **(a)** The contrast-enhanced chest computed tomographic scan revealed a tracheal mass occupying a size of ~14 mm × 23 mm located at the T2 and T3 vertebrae; **(b)** A coronal computed tomographic scan revealed the tracheal mass; **(c)** A bronchoscopy showed a protruding tumor in the middle and lower trachea with nearly total tracheal occlusion. **(d)** After a partial tumor was removed via bronchoscopy with argon knife and cryotherapy, the tracheal lumen is more obvious patency than before.

After admission, a computed tomography scan of her chest revealed a tracheal mass occupying a size of ~14 mm × 23 mm located at the T2 and T3 vertebrae (Figures 1a,b). A fiberoptic bronchoscopy showed a protruding tumor in the middle and lower trachea with nearly total tracheal occlusion (Figure 1c). The lumen was severely narrowed, and the distal end was inaccessible. We removed part of the tumor via bronchoscopy with an argon knife and cryotherapy for symptom relief. After that, the tracheal lumen had more obvious patency than before (Figure 1d), and the ridge of the carina was sharp. Adenoid cystic carcinoma (ACC) was diagnosed after bronchoscopic biopsy. After preoperative examinations including lung function tests, electrocardiography, echocardiography were performed, surgical contraindications were excluded, and informed consent from the patient was obtained, we decided to perform ECMO-assisted uniportal thoracoscopic tracheal tumor resection and tracheoplasty.

### Surgical Procedures

With the patient in the supine position, left subclavian vein catheterization was performed after induction anesthesia. After heparinization, venovenous (VV) ECMO intubation of the right femoral vein-right internal jugular vein was established rapidly. Then the patient was placed in the left lateral decubitus position. A single incision of ~4 cm was made in an intercostal space on the right midaxillary line at the fourth intercostal space (**Figure 3a**). The incision was protected with a wound protector. An endoscope was used as our camera and was placed in the posterior site of the wound (**Figure 3b**). After endoscopic access, a mass ~2 cm in diameter was visible in the upper tracheal segment 1 cm from the carina. The mass was ulcerated and raised, and the membrane was slightly externally invaded. Enlarged lymph nodes were found adjacent to the trachea 4 cm from the glottis.

**Figure 2:**
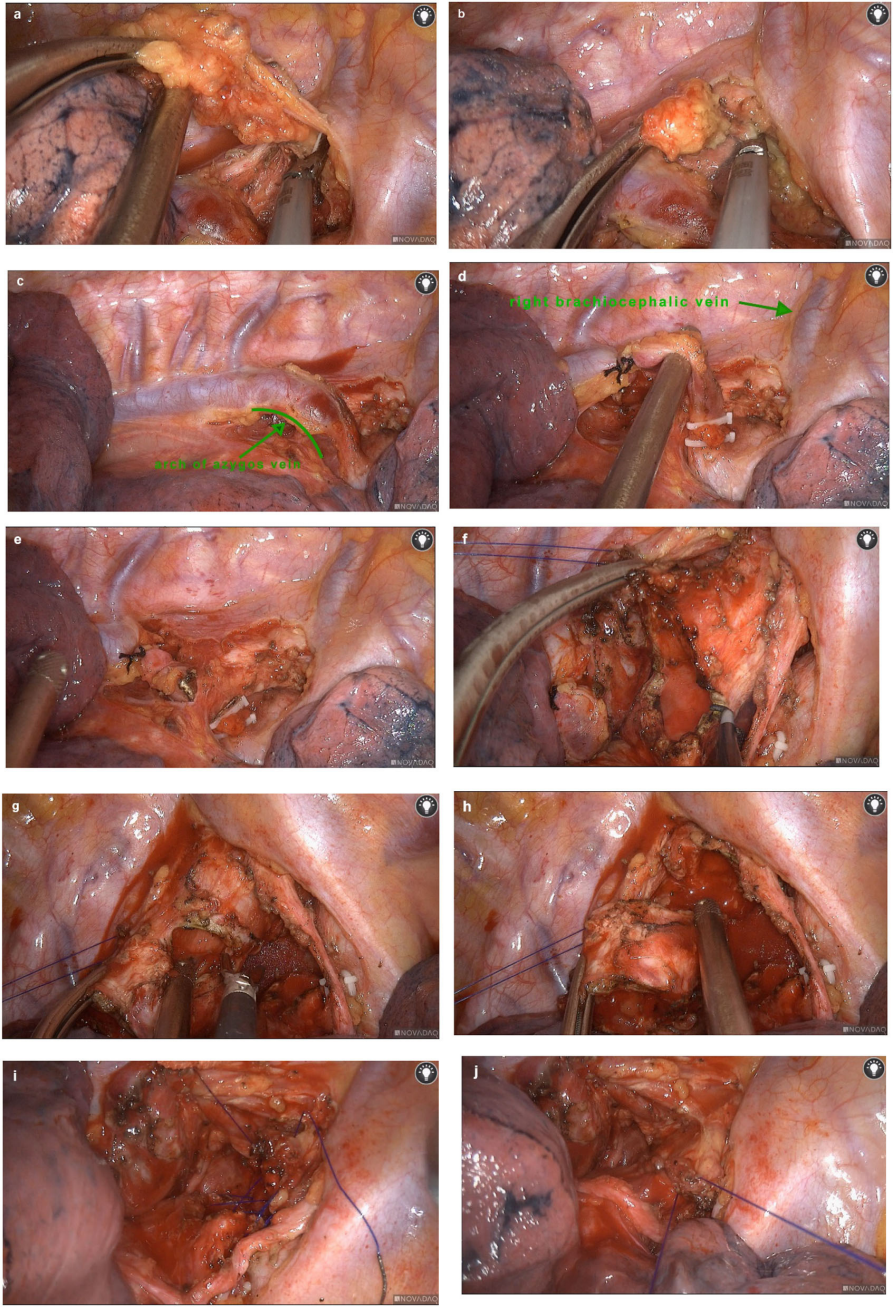
Surgical procedures. We used an electric hook and an ultrasonic knife to clean up the upper paratracheal lymph nodes **(a,b)**. The azygos vein was exposed **(c)**, ligated **(d)** and divided **(e)**. After exposing the intact upper trachea, we cut the end of the tumor in the upper trachea **(f)**. Then we cut the the trachea at the beginning of the tumor **(g)** to remove the tracheal segment where the tumor was located completely **(h)**. Absorbable sutures were used for continuous sutures to connect the tracheal margins at both ends **(i,j)**.

We used electric hook to break the adhesions from the chest cavity and open the mediastinal pleura. The upper paratracheal lymph nodes dissection was performed (Figures 2a,b). Then we isolated the azygos vein (Figure 2c), ligated (Figure 2d) and divided (Figure 2e) it. After exposing the intact superior segment of trachea, we used an electric hook and an ultrasonic knife to cut the end of the tracheal tumor (Figure 2f) and the beginning of the tumor (Figure 2g) in the superior segment of trachea where the tumor was located (Figure 2h). A tracheal tumor specimen was removed and sent for pathological examination. Then, two 3–0 absorbable sutures were used for continuous sutures to connect the tracheal margins at both ends (Figures 2i,j). The anastomosis was checked with the underwater method after the endotracheal tube was placed above the anastomosis. No air leakage was noted after reinforcement under 25-cm H_2_O ventilatory pressure. After adequate hemostasis by porcine-derived fibrin adhesive and composite microporous polysaccharide hemostatic powder, we placed one chest tube as a drainage tube (Main surgical procedure video are given in Supplementary Video 1).

At the same time, ECMO was discontinued and we performed endotracheal intubation to assist with breathing. To relieve the tension after tracheoplasty, a lower jaw to upper sternal socket was secured with 4–0 silk sutures to maintain a low head position. The whole procedure took 232 min with an intraoperative blood loss of ~100 mL. During the surgery, the VV-ECMO oxygen supply was satisfactorily maintained, and the surgical field was clearly revealed.

## Results

**Figure 3:**
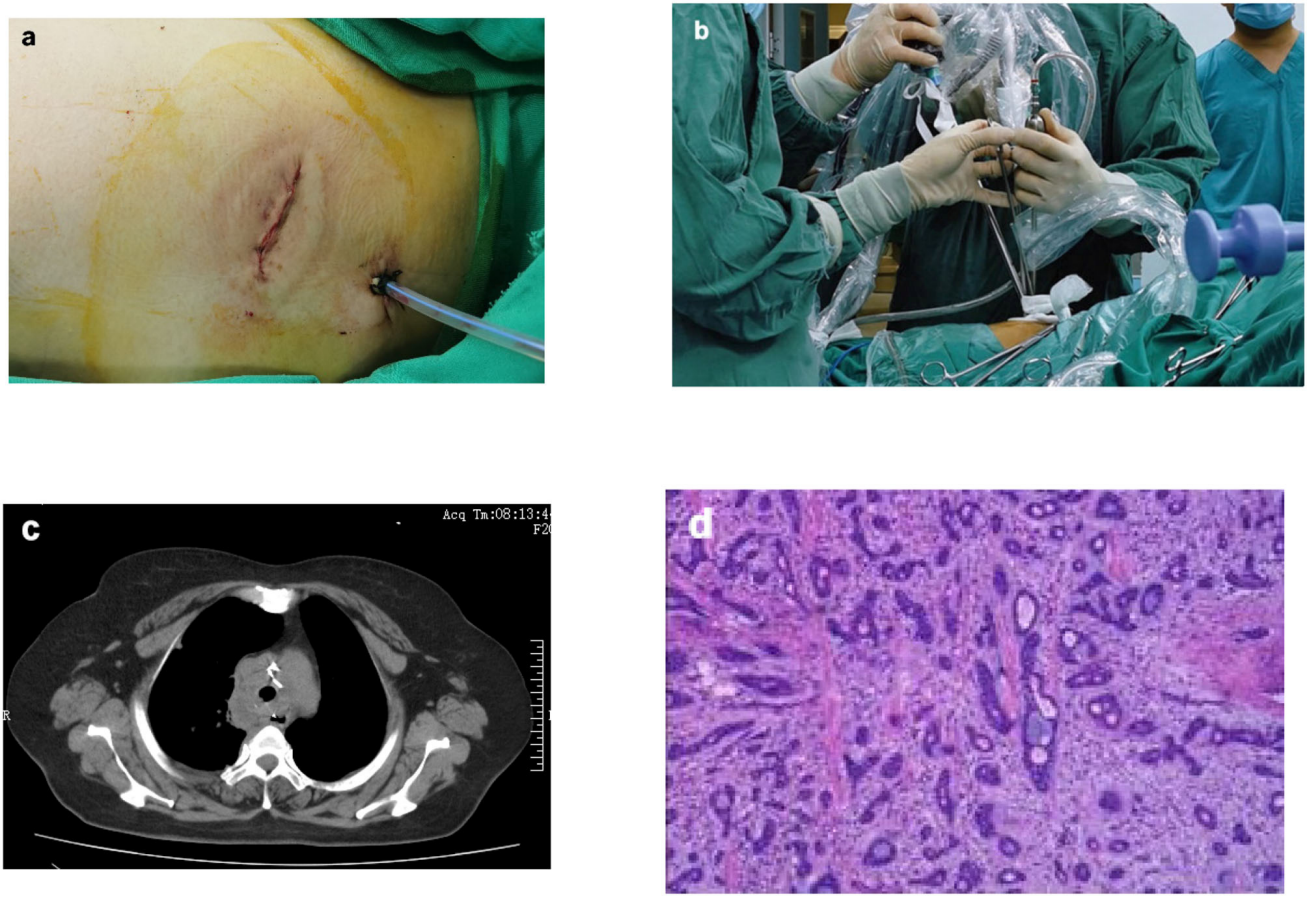
Surgical photos and postoperative examination of the patient. The surgical incision was ~4 cm **(a)**; The endoscope and surgical instruments were introduced via the same incision **(b)**; Postoperative CT revealed that the trachea was patent **(c)**; Postoperative pathologica results revealed adenoid cystic carcinoma (ACC) **(d)**.

**Figure 4:**
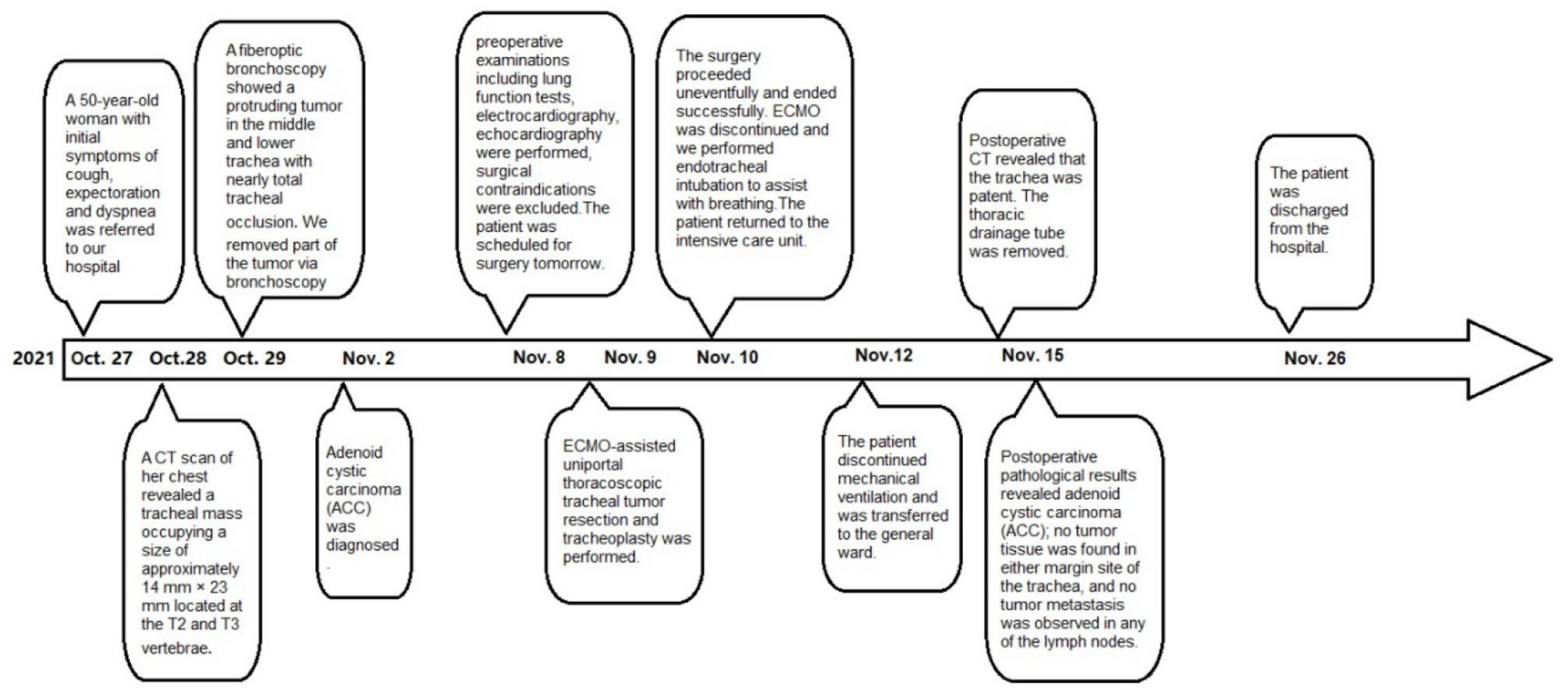
A timeline with relevant data from the episode of care.

The patient returned to the intensive care unit and recovered well after the surgery. The patient discontinued mechanical ventilation on the third postoperative day and was transferred to the general ward. The thoracic drainage tube was removed on the fifth postoperative day, and her head was kept low for 2 weeks. Postoperative CT revealed that the trachea was patent (Figure 3c). She was discharged from the hospital 17 days after the operation. Postoperative pathological results revealed adenoid cystic carcinoma (ACC) (Figure 3d); no tumor tissue was found in either margin site of the trachea, and no tumor metastasis was observed in any of the lymph nodes. One month after discharge, the patient was admitted to hospital for review and consolidation therapy. The results of CT and fiberoptic tracheoscopy review were satisfactory, and the patient was discharged uneventfully after 1 week of tongguanteng injection and cefoperazone sulbactam treatment. A timeline for the entire process with relevant data from the episode of care is shown in Figure 4.

## Discussion

Tracheal surgery is difficult in two aspects: anesthesia and surgery. On the one hand, conventional tracheal surgery generally involves the application of two endotracheal intubation or high-frequency ventilation catheters, simultaneously crossing the surgical field, and it has a high requirement for anesthesiologist cooperation. At the same time, it increases the difficulty of tracheal anastomosis. It is necessary not only to maintain the patient's oxygen supply but also to minimize the interference of intubation with the surgery. Hypoxia can cause multiple organ malfunctions during surgery, which leads to serious complications and even surgical failure. Thus, using conventional ventilation has difficulty obtaining satisfactory results. Shuben Li's group performed the video-assisted transthoracic surgery resection of a tracheal mass and reconstruction of trachea in a Non-intubated patient with spontaneous breathing for the first time in 2016 (8). Their procedure is extremely innovative. However, the surgical team must have extensive experiences in Non-intubated anesthesia with spontaneous breathing and are able to perform active intervention along with endotracheal intubation or thoracoscopic intubation when facing surgery-related complications. This tracheal procedure under spontaneous breathing is demanding for the anesthesiologist and strict grasp of the indications. During the procedure, there is a possibility of tracheal intubation again, posing challenges for stability and coherence of tracheal surgery (8). In our view, ECMO is a perfect alternative during cardiothoracic surgeries because it provides respiratory and hemodynamic support, which can partially or completely replace heart and lung function over a short period of time. The patient's oxygenation and carbon dioxide discharge can be guaranteed. Under this condition, ECMO can be used to maintain the oxygen supply without tracheal intubation or high-frequency ventilation catheter manipulation during the procedure. The first case report of an ECMO-assisted intratracheal tumor resection and carina reconstruction was published in 2019, which was an open surgery (7). On the other hand, tracheal resection is traditionally approached with a right lateral thoracotomy or median sternotomy. Studies on thoracoscopic tracheal resection is scarce, and most of them used multiple incision-ports. The first case report of a uniportal thoracoscopic tracheal resection was published in 2016 (9). We have also performed several ECMO-assisted thoracoscopic tracheal tumor resection procedures successfully. These cases have greatly increased our experience.

Based on the extensive experience, we chose to combine ECMO and uniportal thoracoscopy innovatively. We used a uniportal incision at the fourth intercostal space in the axillary midline and broke the convention of open operation in tracheal surgery, which would minimize the surgical trauma of patients. The intraoperative manipulation was only 180 min in duration. We confirmed the feasibility of ECMO-assisted uniportal thoracoscopic tracheal tumor resection and tracheoplasty. This surgical approach places higher operational technical requirements on the operator but reduces the risk of the patient choking when traditionally transitioning the endotracheal tube. The operation time is significantly shorter, and the surgical approach was more minimally invasive. In addition, it reduces postoperative pain in patients and speeds up the recovery time (10).

In conclusion, as more thoracoscopic surgeries occur, uniportal thoracoscopic tracheal resection could become a common option for patients. An increasing number of tracheal tumors will be treated with minimally invasive surgery. ECMO-assisted uniportal thoracoscopic tracheal resection provides a new idea and method for colleagues. We believe that our efforts will eventually allow more patients to benefit from this type of minimally invasive surgery.

## Author's Note

This article describes a new surgical approach for tracheal tumors. Uniportal thoracoscopic tracheal resection has been reported previously, and ECMO-assisted tracheal resection has also been reported. This article is the first reported case of ECMO-assisted uniportal thoracoscopic tracheal tumor resection and tracheoplasty, which is safe and less invasive. It is therefore a surgical approach worth referring to.

## Data Availability Statement

The original contributions presented in the study are included in the article/Supplementary Material, further inquiries can be directed to the corresponding author.

## Ethics Statement

Written informed consent was obtained from the individual(s) for the publication of any potentially identifiable images or data included in this article.

## Author Contributions

Z-HL drafted and revised manuscript. BD, C-LW, S-HL, and FL provided writing guidance. YQ, BW, and Y-LS designed and participated in this surgery. YQ provided the overall supervision and guidance. All authors have read and approved the final manuscript. All authors contributed to the article and approved the submitted version.

## Conflict of Interest

The authors declare that the research was conducted in the absence of any commercial or financial relationships that could be construed as a potential conflict of interest.

## Publisher's Note

All claims expressed in this article are solely those of the authors and do not necessarily represent those of their affiliated organizations, or those of the publisher, the editors and the reviewers. Any product that may be evaluated in this article, or claim that may be made by its manufacturer, is not guaranteed or endorsed by the publisher.
